# Characterization and priming of equine muscle-derived mesenchymal stem cells to enhance their anti-inflammatory and immunomodulatory profiles

**DOI:** 10.3389/fvets.2025.1741322

**Published:** 2026-01-12

**Authors:** Muhammad A. Shahid, Albert Sole Guitart, François-René Bertin, Olivier Simon, Justine Ceusters, Didier Serteyn, Deanne J. Whitworth

**Affiliations:** 1 The University of Queensland, Brisbane, QLD, Australia; 2 The University of Adelaide, Adelaide, SA, Australia; 3 Universite de Liege, Liège, Belgium

**Keywords:** anti-inflammatory, horse, immunomodulation, MSCs, priming

## Abstract

A minimally invasive microbiopsy-based method for the isolation of mesenchymal stem cells (MSCs) from equine skeletal muscle (M-MSCs) provides a readily accessible source of MSCs for clinical applications. We examined the expression of genes associated with immunomodulation and anti-inflammatory pathways, in addition to those of growth factors and the major histocompatibility complex (MHC) molecules I and II, at constitutive levels and after priming with inflammatory cytokines, an immunostimulant, and heat-shocking. While there was notable variation between the M-MSCs from each of the horses in their constitutive expression of many of the genes examined, and in their responses to the different priming methods, priming with TNF-*α* and IFN-*γ* increased the expression of genes associated with anti-inflammatory pathways, immunomodulation, and tissue repair. M-MSCs from all horses constitutively expressed *MHC-I* and lacked expression of *MHC-II*; only heat-shocking induced the expression of *MHC-II*. The responses to priming, together with their ease of harvesting, supports further investigation into the use of M-MSCs as a therapy for inflammatory and immune-mediated conditions in the horse. However, due to the variability between M-MSCs from different individuals, characterization of the cells before autologous administration, and the selection of those cells most fit-for-purpose in the case of allogeneic transfer, is recommended.

## Introduction

Mesenchymal stem cells (MSCs) have been isolated from various tissues such as bone marrow, adipose tissue, placenta, intestine, nervous tissue and heart (1, 2). MSCs are clinically important due to their ability to self-renew, differentiate into a variety of different cell types, and regulate inflammatory and immunomodulatory processes (3–6). The secretome of MSCs, which contains a variety of bioactive substances such as growth factors, cytokines, chemokines and microvesicles determines their paracrine effects which, in turn, are determined and moderated by the cellular milieu (3, 4, 7–10). Many investigations have shown that altering biological, pharmacological, and/or biophysical parameters affects MSC differentiation capabilities and the bioactive factors that they secrete, which potentially increases their therapeutic potential (11, 12). This process of priming, or licensing, of MSCs can be used to up-regulate their *in vivo* therapeutic functions by increasing their anti-inflammatory and immunomodulatory profiles, angiogenic properties, ability to migrate and prolonging their survival (13–15). Various treatments such as the application of cytokines, growth factors, pharmacological drugs, biomaterials and maintenance under different culture conditions, including hypoxia, have been used to prime MSCs (16–20). Significantly, different priming techniques have different effects in terms of the cytokines, chemokines and growth factors produced by the MSCs, which ultimately affects their role in modulating inflammation and immune responses (21–23).

The most common sources for equine MSCs are bone marrow and adipose tissue, both of which require sedation of the horse and an invasive extraction of tissue. However, a minimally invasive microbiopsy-based method for the isolation of MSCs from equine skeletal muscle, that does not require sedation, has been developed in which the ease of harvesting, and rapid expansion of the cells, combine to make these muscle-derived MSCs (M-MSCs) very appealing for autologous and allogeneic therapies (24). Given that the strongest potential for MSCs as a cell-based therapy lies in their abilities to dampen inflammation and modulate the immune response (25–27), we sought to better understand the anti-inflammatory and immunomodulatory potential of the M-MSCs by examining their responses to the inflammatory cytokines tumor necrosis factor-*α* (TNF-α) and interferon-*γ* (IFN-γ), the immunostimulant polyinosinic:polycytidylic acid (poly(I:C)) and by heat-shocking. TNF-α and IFN-γ are inflammatory cytokines produced by activated T cells (28) that have been shown to improve the anti-inflammatory and immunomodulatory functions of MSCs across a number of species, including horse and dog (22, 29–33). Poly(I:C) is an immunostimulant used to simulate infection by double-stranded RNA viruses (34). It binds to the Toll-like receptor-3 (TLR-3) (35) which is expressed by B lymphocytes, dendritic cells and macrophages (36), and when used to prime MSCs from horse and human has been shown to improve their immunomodulatory activity (37–40). Exposing cells to elevated temperatures induces the production of heat-shock proteins which is an evolutionarily highly conserved response to cellular stress (41). In human, dog, and rat, heat-shock proteins also play an important role against inflammatory conditions such as acute lung injury, acute spinal cord trauma and osteoarthritis by inducing the expression of anti-inflammatory cytokines (42–45). Thus, we explored the possibility of increasing the production of anti-inflammatory factors by the M-MSCs via heat-shocking.

## Materials and methods

All of this research was performed at The University of Queensland (UQ) using established cell lines (provided by co-authors Justine Ceusters and Didier Serteyn) and so UQ Animal Ethics approval was not required.

The microbiopsy procedures that were performed on horses to isolate the M-MSCs that were subsequently donated for this study were approved by the Animal Ethical Commission of the University of Liège.

### Culture and priming of the M-MSCs

Procedures for the sampling and culturing of the M-MSCs have been described by Ceusters et al. (24). Briefly, microbiopsies were taken from the triceps brachii muscle of horses under local anesthesia and maintained in culture in DMEM/F12 culture medium with HEPES and glutamine (Lonza, Verviers, Belgium), 20% fetal bovine serum (FBS; Fisher Scientific, Aalst, Belgium), 100 U/mL penicillin-100 μg/mL streptomycin (Lonza) and 2.5 μg/mL amphotericin B (Lonza) for approximately 13 days until sufficient M-MSCs had grown out from the muscle, at which time they were harvested and subsequently characterized to confirm their identity as MSCs (24).

The M-MSCs used in this study were derived from three horses: Horse 1 (mare, 19 years old, French trotter), Horse 2 (gelding, 19 years old, Standardbred) and Horse 3 (mare, 18 years old, French trotter). The M-MSCs were maintained in DMEM/F-12 with GlutaMAX (Gibco, ThermoFisher Scientific, Australia) supplemented with 20% FBS (Gibco), and 0.1 mM non-essential amino acid solution (NEAA; Gibco). M-MSCs at passage 8 were used for all experiments. At passage 8, M-MSCs showed no indications of entering senescence, with cells maintaining their population doubling time and sharply-defined stellate shape. M-MSCs were seeded at a density of 1 × 10^4^ cells per T25 culture flask and maintained until they reached 50 to 60% confluence at which time they were subjected to each of the three treatments – TNF-*α* and IFN-*γ*, poly(I:C), and heat-shocking – as described below.

For each horse, M-MSCs allocated to the Cytokine (Cy) group were primed with 50 ng/mL of equine TNF-*α* and 50 ng/mL of equine IFN-*γ* (Kingfisher Biotech, USA) for 72 h. A pilot study in which TNF-α and IFN-γ were used at three concentrations of 5 ng/mL, 10 ng/mL, and 50 ng/mL of medium (data not shown) demonstrated that 50 ng/mL induced the most significant upregulation in the expression of anti-inflammatory and immunomodulatory factors as compared to the other concentrations and so was the concentration used for the final experiments. For poly(I:C) priming, M-MSCs were cultured with poly(I:C) (Sigma-Aldrich, Merck-Millipore Australia) at 25 μg/mL of medium for 24 h (40). Heat-shock (H) priming was performed for 1 h at 43^ο^C followed by culture at 37^ο^C for 3 h (10, 32). Control (C) M-MSCs for each of the treatment groups were maintained in medium for the duration of the treatments. Each treatment and the controls were performed in triplicate. Total RNA was extracted from each sample to examine the expression of genes associated with immunomodulation and anti-inflammatory pathways, growth factors, Toll-like receptors (TLRs) that are involved in the innate immune response and inflammatory pathways, and the major histocompatibility complex (MHC) molecules I and II.

### Quantitative RT-PCR

Total RNA was extracted using the NucleoSpin® RNA kit (Macherey-Nagel GmbH, ThermoFisher Scientific, Australia) and cDNA prepared using the iScript Reverse Transcriptase kit (Bio-Rad Laboratories, Australia). The expression of anti-inflammatory and immunomodulatory factors, growth factors, TLRs and MHCs-I and -II were compared using quantitative RT-PCR (qRT-PCR) on a CFX-96 Real-time PCR Detection System (Bio-Rad Laboratories, Australia) using the SsoAdvanced Universal SYBR Green Supermix (Bio-Rad Laboratories). Data were normalized to the expression of equine *β-ACTIN*. Table 1 lists the validated primers and their product sizes. For each sample, three technical replicates were performed. The cycle parameters were denaturation at 95 °C for 3 min, 45 amplification cycles (95 °C, 10 s; 62 °C, 20 s), and elongation at 75 °C for 1 min. Melt curve analysis was performed over a temperature range of 65–95 °C in 0.5 °C increments for 0.05 s. The relative expression ratios of genes were calculated using the Delta Ct method, as described by Livak et al. (46). For the comparison of treatments, the constitutive levels of expression of the controls have been normalized to 1 and the treatment results are presented relative to the normalized control.

**Table 1:** Equine primers used to analyze the expression of genes in M-MSCs.

Gene	Primers	Sequence (5′ to 3′)	Product size (bp)
*β-ACTIN*	Forward	GATGATGATATCGCCGCGCTC	167
	Reverse	TCGTCGCCCACGTATGAGTC	
*COX-2*	Forward	GATCCTAAGCGAGGTCCAGC	101
	Reverse	AGGCGCAGTTTATGCTGTCT	
*IL-8*	Forward	GCTTTCTGCAGCTCTGTGTGA	190
	Reverse	GCAGACCTCAGCTCCGTTGA	
*IL-6*	Forward	CACCACTGGTCTTTCGGAGT	175
	Reverse	GCTGCTTTTTGCAGTTGGGT	
*VEGF*	Forward	TGCGGATCAAACCTCACCAA	114
	Reverse	GCCCACAGGGATTTTCTTGC	
*LOXL-2*	Forward	TGACTTCCGACCCAAGAACG	155
	Reverse	TGTCCTCCAAGCAGAAGCTG	
*FGF-2*	Forward	CGGCTCTACTGCAAAAACGG	151
	Reverse	GGTTCGCACACACTCCTTTG	
*TLR-2*	Forward	GGACTCTCTTTCTCGGTGCC	131
	Reverse	CTAAGACCCACACCGTCCAC	
*TLR-3*	Forward	ACCTCCCAGCAAACATAACG (90)	179
	Reverse	CTGGAGGTCCAAAATTTCCA	
*TLR-9*	Forward	TTGCCGCGTGAAGGGAC	99
	Reverse	ATGACAGGGAGTGGGAGATGAT	
*TSG-6*	Forward	ACAGGTTGCTTGGCTGACTA	188
	Reverse	TCCACTGCCACGTACTTGAT	
*IL-10*	Forward	GCCTTGTCGGAGATGATCCA (29)	81
	Reverse	TTTTCCCCCAGGGAGTTCAC	
*HO-1*	Forward	CTCCTGCTTGTCTGGAGTCG	114
	Reverse	ACGGCCTCTGACAAATCCTG	
*IDO*	Forward	ATCAAAGAAATTCCGATTATATTCAA	97
	Reverse	TGCGTAGACAAGAAGAAGTTATATCAAT	
*iNOS*	Forward	TGGTGGATGGCCCTGCA (91)	246
	Reverse	ATGTGGGGCTGTTGGTGA	
*MHC-I*	Forward	ACCGTGAGGTCACCCTGA	101
	Reverse	CTCCGTGTCCTGGGTCA	
*MHC-II*	Forward	TCCCTATGCTGGGACTTTTC (29)	111
	Reverse	CGCCAGGCTTCAGATAGAAC	

### Statistical analyses

The expression levels of each of the genes examined in the M-MSCs from each horse and subjected to each treatment were compared with their respective control. The results are presented as mean ± standard error of the mean (SEM). Statistical significance was determined through a one-way ANOVA and post-hoc Tukey test among the treated groups and their controls. Microsoft Excel Microsoft 365 MSO (Version 2,303) and GraphPad7 Prism software (San Diego, CA, USA) were used to analyze the data. Significance is defined as *p* < 0.05.

## Results

### Expression of genes involved in immunomodulation

#### Cyclooxygenase-2 (COX-2)

**Figure 1:**
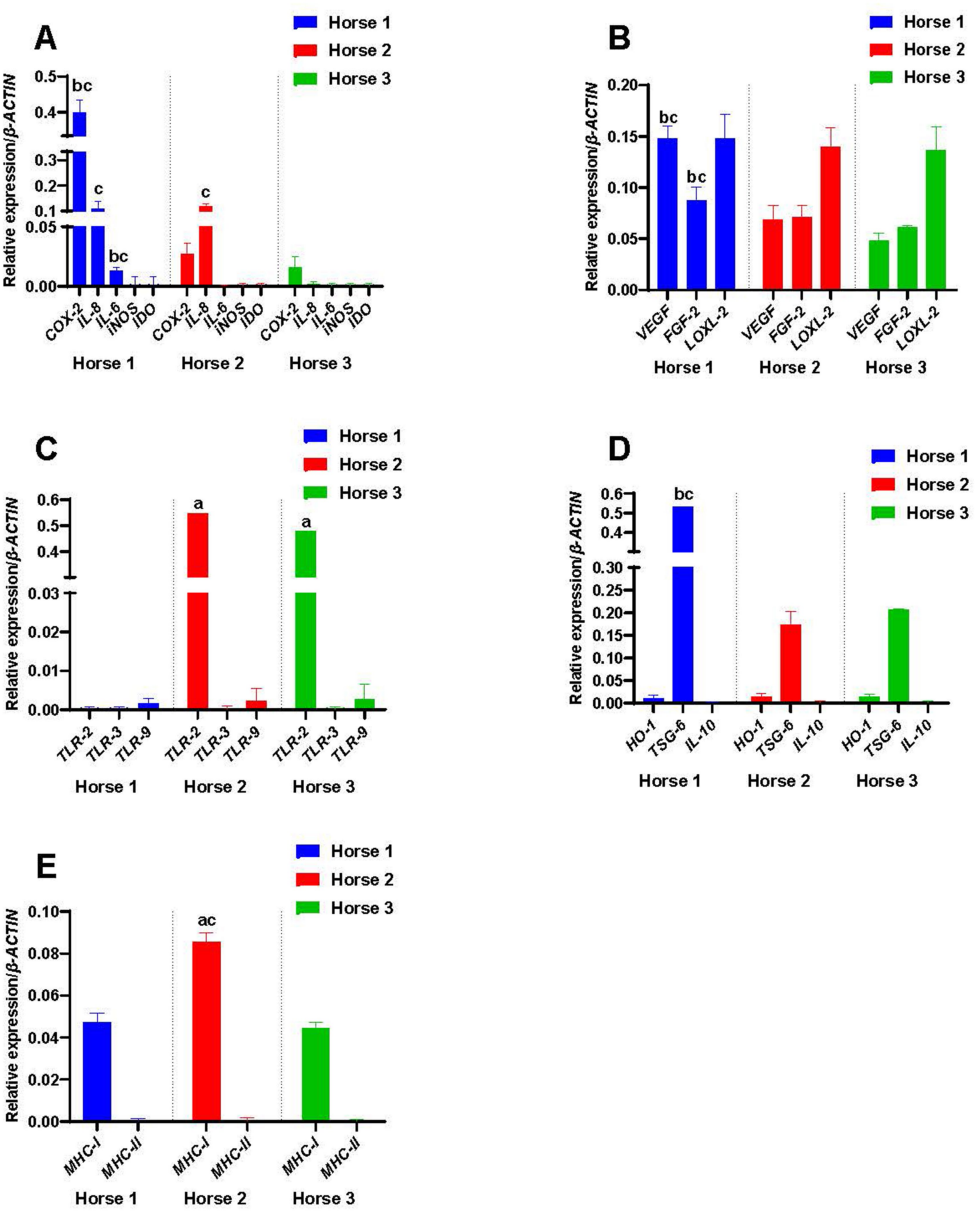
Constitutive expression of **(A)** immunomodulatory factors, **(B)** growth factors, **(C)** toll-like receptors (TLRs), **(D)** anti-inflammatory factors, and **(E)** major histocompatibility complex (*MHC*) molecules. The letter ‘a’ denotes a significant difference (*p < 0.05*) from Horse 1-derived M-MSCs; ‘b’ from Horse 2-derived M-MSCs; and ‘c’ from Horse 3-derived M-MSCs.

**Figure 2:**
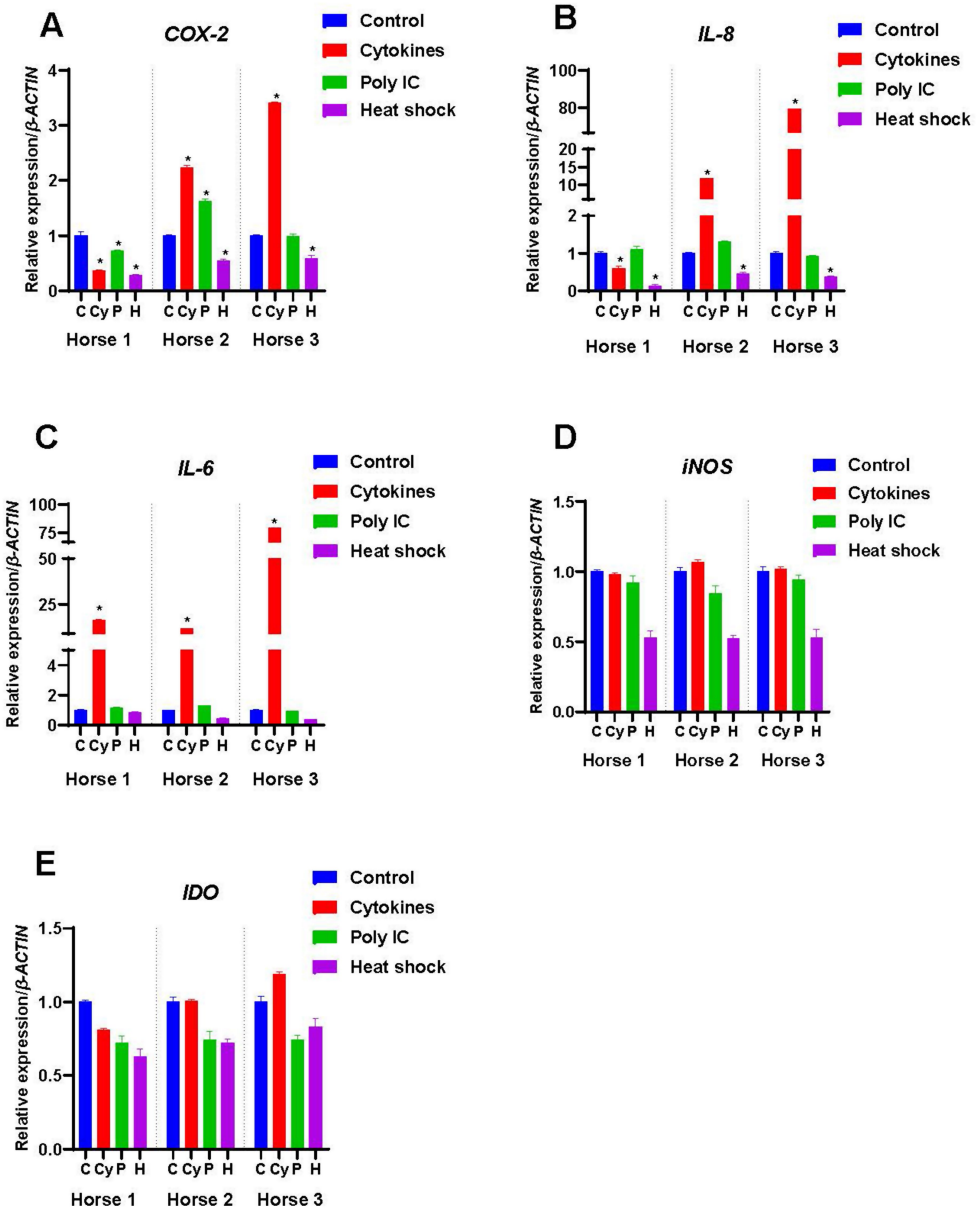
Expression of immunomodulatory factors by M-MSCs from Horses 1, 2 and 3 when primed with inflammatory cytokines TNF-*α* and IFN-*γ* (Cy), poly(I:C) (P), and heat-shocking (H) as compared to expression in control unprimed M-MSCs (C). The constitutive levels of the controls were normalized to 1 and the treatment results are presented relative to the normalized control. An asterisk (*) indicates a significant difference (*p < 0.05*) from the respective control.

The unprimed M-MSCs from all three horses expressed *COX-2* constitutively although levels varied markedly between individuals (Figure 1A), with Horse 1 expressing significantly higher levels of *COX-2* compared to the other two horses (*p* < 0.05). Cytokine priming significantly decreased the expression of *COX-2* in Horse 1-derived M-MSCs as compared to the control (*p* < 0.05), while it significantly increased the expression of *COX-2* in M-MSCs from Horse 2 and Horse 3 as compared to their respective controls (*p* < 0.05) (Figure 2A). Poly(I:C) priming reduced the expression of *COX-2* in Horse 1-derived M-MSCs (*p* < 0.05), while it significantly increased the expression of *COX-2* in M-MSCs from Horse 2 (*p* < 0.05) and Horse 3-derived M-MSCs showed no significant change in *COX-2* expression as compared to the control (Figure 2A). Heat-shocking significantly decreased the expression of *COX-2* in M-MSCs from all three horses as compared to their respective controls (*p* < 0.05) (Figure 2A).

#### Interleukin-8 (IL-8)

*IL-8* was expressed by unprimed M-MSCs from Horses 1 and 2, but not by those from Horse 3 (Figure 1A). Cytokine priming significantly decreased the expression of *IL-8* in Horse 1-derived M-MSCs (*p* < 0.05), while it significantly increased the expression of *IL-8* in the M-MSCs from Horses 2 (*p* < 0.05) and 3 (*p* < 0.05) (Figure 2B). Poly(I:C) priming did not produce any significant change in the expression of *IL-8* in the M-MSCs from any of the three horses while heat-shocking significantly decreased the expression of *IL-8* in M-MSCs from Horses 2 (*p* < 0.05) and 3 (*p* < 0.05) below their constitutive levels (Figure 2B).

#### Interleukin-6 (IL-6)

*IL-6* was expressed by M-MSCs from Horse 1, but not by those from Horses 2 & 3 (Figure 1A). Cytokine priming significantly increased (*p* < 0.05) the expression of *IL-6* in M-MSCs from all three horses while poly(I:C) did not significantly affect the expression of *IL-6* in M-MSCs from any of the horses. Similarly, there was no significant effect on *IL-6* expression in M-MSCs from any of the horses in response to heat-shocking (Figure 2C).

#### Inducible nitric oxide synthase (iNOS)

*iNOS* was not expressed constitutively by M-MSCs from any of the three horses (Figure 1A), and cytokine, poly(I:C) and heat-shocking treatments had no significant effects on the levels of *iNOS* expression (Figure 2D).

#### Indoleamine 2,3-dioxygenase

*IDO* was also not expressed constitutively by M-MSCs from any of the three horses (Figure 1A). Neither cytokine, poly(I:C) nor heat-shocking treatments had any significant effects on the levels of *IDO* expression (Figure 2E).

### Expression of growth factors

#### Vascular endothelial growth factor (VEGF)

**Figure 3:**
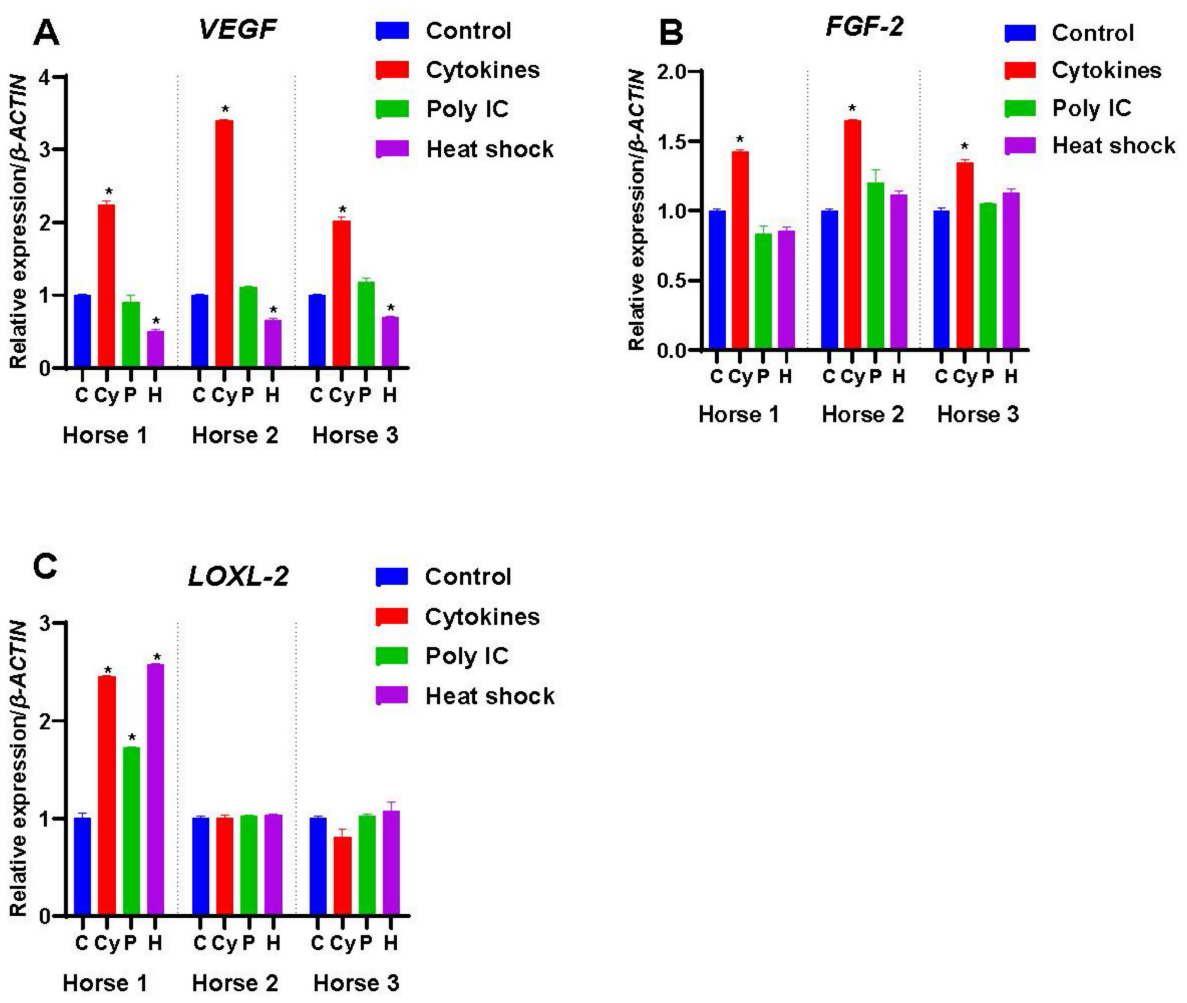
Expression of growth factors by M-MSCs from Horses 1, 2 and 3 when primed with inflammatory cytokines TNF-α and IFN-γ (Cy), poly(I:C) (P), and heat-shocking (H) as compared to expression in control unprimed M-MSCs (C). The constitutive levels of the controls were normalized to 1 and the treatment results are presented relative to the normalized control. An asterisk (*) indicates a significant difference (*p < 0.05*) from the respective control.

M-MSCs from all three horses expressed *VEGF* constitutively, with the highest levels detected in Horse 1 (*p* < 0.05) (Figure 1B). Cytokine priming significantly increased (*p* < 0.05) the expression of *VEGF* in M-MSCs from all three horses as compared to their controls (Figure 3A), while priming with poly(I:C) had no significant effect on the expression of *VEGF* (Figure 3A). In contrast, heat-shock treatment significantly decreased (*p* < 0.05) the expression of *VEGF* in all three M-MSC samples (Figure 3A).

#### Fibroblast growth factor-2 (FGF-2)

*FGF-2* was expressed constitutively by the M-MSCs from all three horses (Figure 1B). M-MSCs from Horse 1 had significantly higher expression of *FGF-2* than those from Horse 2 and Horse 3 (*p* < 0.05). Cytokine priming significantly increased (*p* < 0.05) the expression of *FGF-2* in all horses, while poly(I:C) and heat-shocking each had no significant effect (Figure 3B).

#### Lysyl oxidase homolog-2 (LOXL-2)

M-MSCs from all three horses expressed *LOXL-2* at very similar levels (Figure 1B), but only Horse 1-derived M-MSCs showed effects from treatments with cytokines, poly(I:C) and heat-shocking with expression of *LOXL-2* increasing significantly (*p* < 0.05) (Figure 3C).

### Expression of toll-like receptors

#### Toll-like receptor-2 (TLR-2)

**Figure 4:**
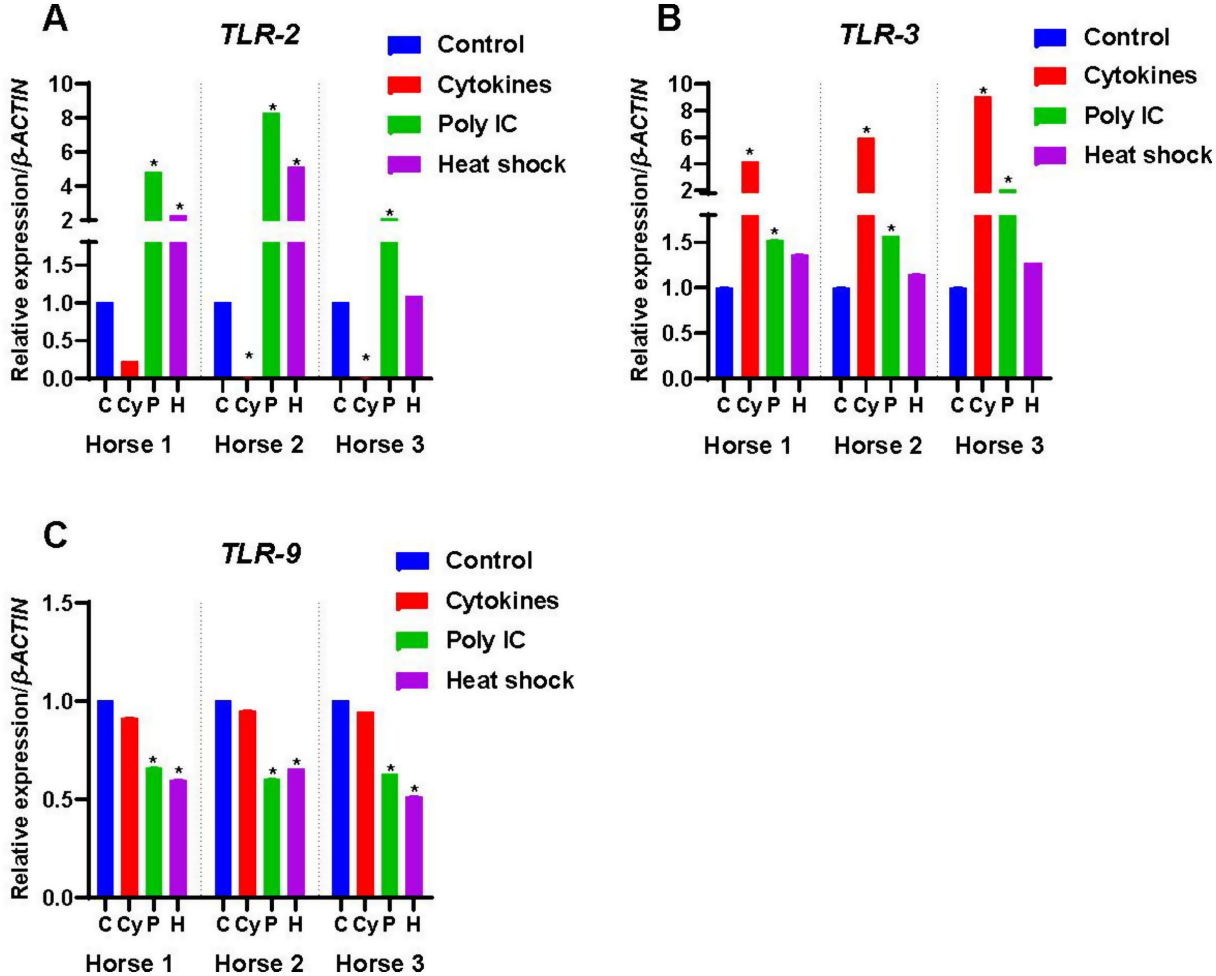
Expression of Toll-like receptors by M-MSCs from Horses 1, 2 and 3 when primed with inflammatory cytokines TNF-α and IFN-γ (Cy), poly(I:C) (P), and heat-shocking (H) as compared to expression in control unprimed M-MSCs (C). The constitutive levels of the controls were normalized to 1 and the treatment results are presented relative to the normalized control. An asterisk (*) indicates a significant difference (*p < 0.05*) from the respective control.

Expression of *TLR-2* was detected in M-MSCs from Horses 2 and 3 but was not expressed by Horse 1 (Figure 1C). Cytokine priming significantly decreased (*p* < 0.05) the expression of *TLR-2* in M-MSCs from Horses 2 and 3 (Figure 4A). While poly(I:C) significantly upregulated (*p* < 0.05) the expression of *TLR-2* in all three horses, transcript levels in Horse 1 were still below the threshold for gene expression (Figure 4A). Heat-shocking increased the expression of *TLR-2* in Horses 1 and 2 (*p* < 0.05), but again transcript levels in Horse 1 remained below the threshold for gene expression (Figure 4A). Heat-shocking had no significant effect on expression levels in Horse 3 (Figure 4A).

#### Toll-like receptor-3 (TLR-3)

*TLR-3* was not expressed constitutively by M-MSCs from any of the horses (Figure 1C). Both cytokine and poly(I:C) priming significantly increased (*p* < 0.05) the expression of *TLR-3* in all three horses while heat-shocking had no significant effect (Figure 4B).

#### Toll-like receptor-9 (TLR-9)

*TLR-9* was expressed constitutively by M-MSCs from all three horses (Figure 1C). Although cytokine priming had no significant effect on the expression of *TLR-9*, poly(I:C) and heat-shock priming both significantly downregulated (*p* < 0.05) the expression of *TLR-9* in M-MSCs from all the horses (Figure 4C).

### Expression of anti-inflammatory genes

#### Haeme oxygenase-1 (HO-1)

**Figure 5:**
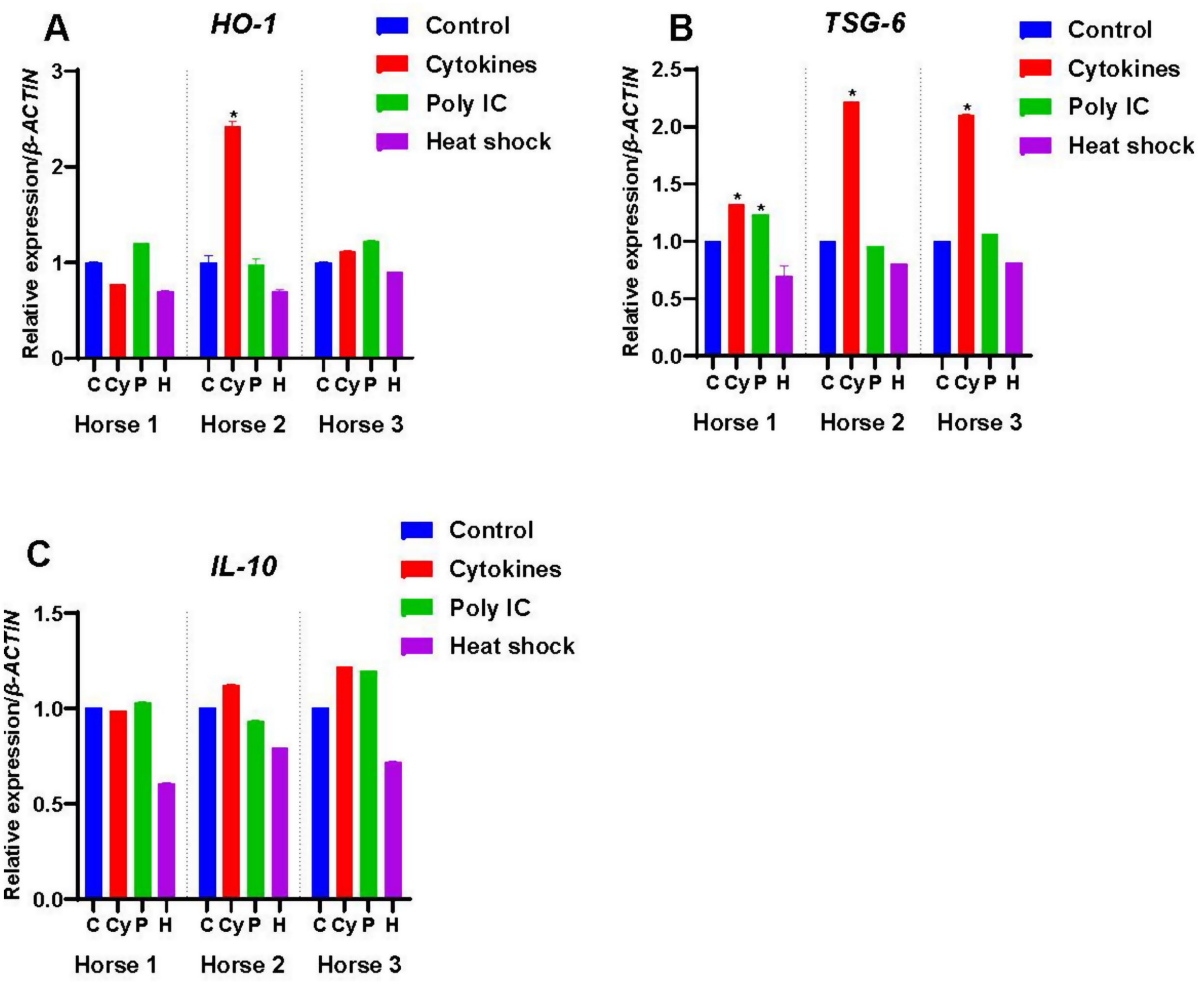
Expression of anti-inflammatory factors by M-MSCs from Horses 1, 2 and 3 when primed with inflammatory cytokines TNF-α and IFN-γ (Cy), poly(I:C) (P), and heat-shocking (H) as compared to expression in control unprimed M-MSCs (C). The constitutive levels of the controls were normalized to 1 and the treatment results are presented relative to the normalized control. An asterisk (*) indicates a significant difference (*p < 0.05*) from the respective control.

*HO-1* was constitutively expressed at low levels by M-MSCs from all the horses (Figure 1D). Of the three treatments across the three horses, only the cytokine priming of M-MSCs from Horse 2 showed a significant response with an upregulation in expression of *HO-1* (*p* < 0.05) (Figure 5A).

#### Tumor necrosis factor-*α*-stimulated gene-6 (TSG-6)

M-MSCs from all three horses expressed *TSG-6* constitutively (Figure 1D), with M-MSCs from Horse 1 showing significantly higher levels of *TSG-6* expression compared to the M-MSCs from Horses 2 and 3 (*p* < 0.05). Cytokine priming significantly upregulated (*p* < 0.05) the expression of *TSG-6* in the M-MSCs from all the horses (Figure 5B). Poly(I:C) significantly increased *TSG-6* expression in Horse 1 only (*p* < 0.05), while none of the horses showed a significant change in expression in response to heat-shocking (Figure 5B).

#### Interleukin-10 (IL-10)

*IL-10* was not expressed by M-MSCs from any of the horses (Figure 1D), and priming with cytokines, poly(I:C) and heat-shocking had no significant effect upon expression levels (Figure 5C).

### Expression of genes involved in immunogenicity

#### Major histocompatibility complex-I (MHC-I)

**Figure 6:**
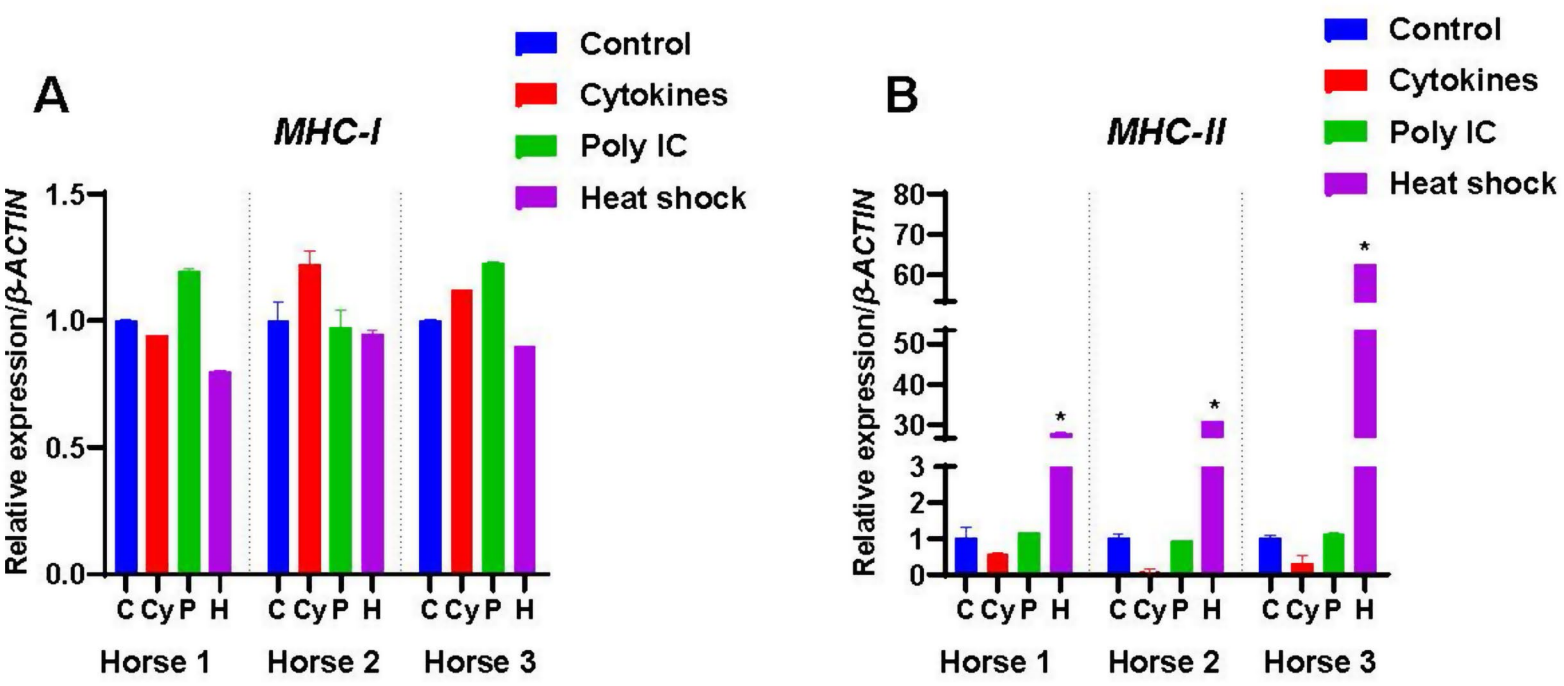
Expression of MHC-I and MHC-II by M-MSCs from Horses 1, 2 and 3 when primed with inflammatory cytokines TNF-α and IFN-γ (Cy), poly(I:C) (P), and heat-shocking (H) as compared to expression in control unprimed M-MSCs (C). The constitutive levels of the controls were normalized to 1 and the treatment results are presented relative to the normalized control. An asterisk (*) indicates a significant difference (*p < 0.05*) from the respective control.

As expected, M-MSCs from all the horses expressed *MHC-I*, with the highest levels of expression detected in M-MSCs from Horse 2 (*p* < 0.05) (Figure 1E). However, there was no significant effect of the different treatments on the expression of *MHC-I* in any of the three horses (Figure 6A).

#### Major histocompatibility complex-II (MHC-II)

*MHC-II* was not expressed constitutively by M-MSCs from any of the horses (Figure 1E). Cytokine and poly(I:C) priming had no significant effect on *MHC-II* expression levels (Figure 6B), while heat-shocking significantly increased (*p* < 0.05) the expression of *MHC-II* in all three horses (Figure 6B).

## Discussion

MSCs are a promising treatment option for immune-mediated and inflammatory diseases, and as a stimulant for tissue regeneration through various mechanisms such as angiogenesis, immunosuppressive and anti-inflammatory actions, and the stimulation of cell migration, proliferation, and differentiation (47, 48). The production of bioactive substances such as growth factors, chemokines, and immunomodulatory compounds by MSCs is primarily responsible for their beneficial effects (49).

In this study, we observed that M-MSCs derived from three horses of mixed sex and two different breeds, but of a similar age, exhibited heterogeneity in their constitutive gene expression profiles, before priming with various techniques. This variability was evident in the production of inflammatory factors, anti-inflammatory factors, growth factors, expression of Toll-like receptors and MHC-I. This finding is important because it highlights the inherent heterogeneity of M-MSCs derived from different horses which has previously been described for bone marrow-derived MSCs (BM-MSCs) (50, 51). The significance of this observation lies in several key aspects including variability in therapeutic potential: for instance, the heterogeneity in the production of inflammatory factors, anti-inflammatory factors, and growth factors suggests that unprimed M-MSCs from different horses may respond differently to inflammatory environments *in vivo* and consequently have varying therapeutic effects.

MSCs, including those from the horse, are frequently primed to increase their immunomodulatory and anti-inflammatory properties (16, 23, 37, 41, 52–55). However, there are significant translational barriers, especially in the horse, due to the lack of standardized priming techniques and a limited understanding of the responses of MSCs from different tissues, and individuals, to priming (56). This study examined the effects of three different priming methods on M-MSCs – cytokine stimulation, poly(I:C) activation, and heat shock treatment – all of which have previously demonstrated potential in enhancing MSC immunomodulation and anti-inflammatory functions in horses and humans (16, 23, 52–54, 57–59).

In this study, cytokine priming increased the expression of *HO-1* (Horse 2) and *TSG-6* (all horses) which have been associated with reduced inflammation in rodents and human (59–62), and downregulated the expression of the pro-inflammatory cytokines *COX-2* (Horse 1) and *IL-8* (Horse 1). The downregulation of *COX-2* expression in Horse 1 is in contrast to the other two horses where cytokine priming increased the expression of *COX-2*, which has also been consistently reported in equine BM-MSCs primed with TNF-*α* and IFN-*γ* (16, 52).

Cytokine priming significantly increased the expression of *IL-6* in M-MSCs from all three horses, which is consistent with observations in equine BM-MSCs (16, 23, 52, 54). While the role of *IL-6* in equine immunomodulation is not fully understood, in contrast to its role in human BM-MSCs where it suppresses the proliferation of stimulated T lymphocytes (63), this does not appear to be the case in equine BM-MSCs (64, 65). In our study, only M-MSCs from Horse 1 expressed *IL-6* constitutively, while equine BM-MSCs, adipose tissue-derived MSCs (AT-MSCs) and endometrium-derived MSCs (E-MSCs) all express *IL-6* constitutively, albeit at varying levels (66). Constitutive expression of *IL-6* by human BM-MSCs stimulates their proliferation (63, 67), inhibits their differentiation along the adipogenic and chondrogenic pathways, prevents them from undergoing apoptosis, and increases the rate of their migration in an *in vitro* scratch assay (67). This disparity in constitutive *IL-6* expression between the M-MSCs from different individuals, and in comparison with equine BM-, AT- and E-MSCs, is therefore intriguing given its potential roles, based on human studies, in regulating MSC proliferation, survival and the maintenance of the undifferentiated state.

We also show that cytokine priming significantly upregulates *VEGF* and *FGF-2* in all three horses, and *LOXL-2* in one of the horses (Horse 1). *VEGF* and *FGF-2* play a central role in angiogenesis during tissue regeneration (68, 69), while *FGF-2* is also involved in wound healing and tissue repair (70). *LOXL-2* encodes an enzyme involved in the development of the extracellular matrix by catalyzing the crosslinking of collagen and elastin fibers (71) and, by extension, is involved in maintaining the structural integrity of the connective tissue proper that forms during wound healing (72). This suggests that cytokine-primed M-MSCs may have enhanced tissue repair potential compared to non-primed M-MSCs.

Toll-like receptors (TLRs) are localized to neutrophils, macrophages, dendritic cells and natural killer cells where they play a key role in activating the innate immune response against viral, bacterial and fungal pathogens (73), and their roles in MSC biology is an area of growing research interest. TLRs 2 and 4 are expressed on the cell surface where they recognize lipids and lipoproteins, and lipopolysaccharides, respectively, in microbial membranes (73). TLRs 3 and 9 are expressed intracellularly; TLR-3 recognizes viral double-stranded RNA, while TLR-9 responds to bacterial and viral DNA (73). Human MSCs from BM, AT and umbilical cord blood (UCB) express high levels of *TLR-3* and *TLR-4*, and low levels of *TLR-2* and *TLR-9* (74), while MSCs derived from Wharton’s jelly (WJ) lack expression of *TLR-4* (75). In response to inflammation, BM- and AT-MSCs upregulate *TLR-3* expression, while only the BM-MSCs upregulated *TLR4* (75). Taken together, these data demonstrate that for human MSCs, tissue source influences *TLR* expression profiles and, consequently, MSC responses to inflammation. The data for equine MSCs is less comprehensive, but expression of *TLR-3* in equine MSCs after priming with poly(I:C) improves the bactericidal activity of the MSCs (76), and their immunomodulatory ability in decreasing T lymphocyte proliferation (37). In our study, cytokine- and poly(I:C)-primed M-MSCs from all three horses exhibit increased expression of *TLR-3*. TLR-2 has been implicated in shifting murine BM-MSCs toward a pro-inflammatory phenotype (77). In our study, cytokine priming significantly decreased the expression of *TLR-2* in Horses 2 and 3. Expression of *TLR-9* by human BM-MSCs induces the production of pro-inflammatory cytokines (78, 79). In our M-MSCs, none of the treatments increased the expression of *TLR-9* above constitutive levels in any of the horses which, in combination with the upregulation of the anti-inflammatory *TLR-3* in all of the horses, and a downregulation of the pro-inflammatory *TLR-2* in horses 2 and 3, supports further investigation into the use of cytokine-primed M-MSCs in managing inflammation in the horse.

Human and veterinary clinical interest in MSCs has been principally directed at the use of allogeneic MSCs as a readily accessible, off-the-shelf supply of MSCs. The advantage of allogeneic cells over the use of autologous cells is that fully characterized cells are available immediately to treat acute injuries or conditions, whereas the collection and expansion in culture of patient-specific autologous cells takes time, during which inflammation associated with the acute injury or condition can cause further tissue damage. Autologous cells may also not be as effective when collected from aged or diseased individuals (80). MSCs from different species are typically considered immune privileged due to their ability to evade immune detection by allogeneic hosts; however, a more accurate description is that they are immune evasive, exerting their immunomodulatory and anti-inflammatory effects before ultimately being cleared by the host’s immune system (81). This ability to evade immune detection has been largely attributed to low expression of MHC-I, and low or absent expression of MHC-II (81). Equine BM-, AT-, peripheral blood (PB) and amnion-derived MSCs constitutively express *MHC-I* (16, 23, 50–53, 82), as we observed in this study with M-MSCs. Since priming is required to stimulate the immunomodulatory and anti-inflammatory activities of MSCs, we wished to determine if any of the priming methods that we used would induce a change in *MHC-I* expression. Across the different treatments, the expression of *MHC-I* remained unchanged from constitutive levels in all three horses. The effects of specifically cytokine priming on the expression of *MHC-I* by equine BM-MSCs are varied with some studies reporting an increase in *MHC-I* expression levels and others noting no change; however, this may be due to the concentrations of TNF-*α* and IFN-*γ* used, and the duration of priming, in the different studies since Cequier and colleagues (52), and Barrachina et al. (16) both of whom did not see an upregulation of *MHC-I* expression, primed the BM-MSCs with 5 ng/μl of each of TNF-α and IFN-γ for only 12 h, while an earlier study by Barrachina and colleagues (23), primed with 20 ng/μl of TNF-α and 50 ng/μl IFN-γ for 72 h and did observe an increase in *MHC-I* expression. We primed the M-MSCs with 50 ng/μl of each of TNF-α and IFN-γ for 72 h and still did not see an increase in *MHC-I* expression suggesting that in an inflammatory environment *in vivo*, the M-MSCs are perhaps less likely to upregulate MHC-I expression than BM-MSCs.

We did not detect constitutive expression of *MHC-II* by M-MSCs from any of the horses in this study, which is consistent with an analysis on AT- and PB-MSCs (50), and some studies on BM-MSCs, where the expression of *MHC-II* has been found to be absent, present or variable between individual horses (50–52, 82). As with *MHC-I* expression, the effect of priming with TNF-α and IFN-γ on *MHC-II* expression by equine BM-MSCs differs between studies, but appears to be independent of the concentrations of TNF-*α* and IFN-*γ* used, and the length of exposure: for example, both Barrachina and colleagues (16) and Cequier et al. (52) used 5 ng/μl of each of TNF-*α* and IFN-*γ* for 12 h and observed an upregulation, and no change, to *MHC-II* levels, respectively. Whether equine BM-MSCs, or for that matter MSCs from any tissue source, express *MHC-II* constitutively, and/or upregulate its expression in response to inflammatory stimuli, is difficult to predict and this is clearly illustrated in the study by Schnabel and colleagues (51) where the proportion of BM-MSCs that constitutively expressed *MHC-II* for each of 10 thoroughbreds ranged from 0.18%, through 32.42, to 98.35%.

Heat-shocking significantly upregulated the expression of *MHC-II* to detectable levels in all of the horses. Heat stress induces the production of heat shock proteins (HSPs) that function as molecular chaperones, enabling proteins that have become unfolded or misfolded in response to heat stress to refold into their normal, functional state (83). HSPs also function in antigen processing and in the transfer of antigenic peptides to MHC-I and MHC-II molecules (83) which may go some way to explaining the induced expression of MHC-II in the M-MSCs after heat-shocking.

MSCs exert their immunomodulatory effects, in particular the suppression of T cell proliferation, via paracrine signaling by IDO and iNOS (84–89). MSCs from mouse, rat, rabbit and hamster suppress T cell proliferation via iNOS signaling, while in human, monkey, pig, dog and cow this role is performed by IDO (27). Equine BM-MSCs primed with TNF-*α* and IFN-*γ* upregulate their expression of both *IDO* and *iNOS* (16, 23, 52, 54). In a study comparing the responses of equine AT-, CT-, BM- and CB-MSCs to mitogen-stimulated T cells producing high levels of TNF-α and IFN-γ, Carrade and colleagues (65) found that only the BM- and CB-MSCs produced nitric oxide, which is synthesized by iNOS from L-arginine, and none of the MSCs produced IDO when exposed to the stimulated T cells (65). In our study, M-MSCs did not express *IDO* or *iNOS* constitutively, or after priming, similar to AT- and CT-MSCs that do not produce NO or express IDO when stimulated (64, 65). Carrade Holt and colleagues (64) also determined that MSCs from different tissue sources regulate T cell proliferation via different mechanisms, including apoptosis and cell cycle arrest, that are independent of IDO and iNOS activity, and our M-MSCs appear to similarly activate IDO- and iNOS-independent pathways in response to stimulation with TNF-α and IFN-γ.

In summary, our work highlights a key role for cytokine priming in enhancing the anti-inflammatory, immunomodulatory, and regenerative potential of M-MSCs. However, the heterogeneity between M-MSCs from different individuals poses challenges for standardizing M-MSC-based therapies, as the variability in cell properties could lead to inconsistent treatment outcomes. The main limitation of this study is that it examined M-MSCs from three horses; therefore, the optimisation of therapeutic outcomes will require the characterization of M-MSCs from a variety of individuals to guide the application of appropriate priming strategies to ensure the desired M-MSC secretome for the clinical objective.

## Data Availability

The raw data supporting the conclusions of this article will be made available by the authors, without undue reservation.
